# Diannexin Can Ameliorate Acute Respiratory Distress Syndrome in Rats by Promoting Heme Oxygenase-1 Expression

**DOI:** 10.1155/2021/1946384

**Published:** 2021-04-09

**Authors:** Ying-nan Ju, Qi-hang Tai, Guang-xiao Xu, Xiao-qing Zhao, Hai-bin Sun, Wei Gao

**Affiliations:** ^1^Department of Intensive Care Unit, The Third Affiliated Hospital of Harbin Medical University, Harbin, Heilongjiang Province 150081, China; ^2^Department of Anesthesiology, The Second Affiliated Hospital of Harbin Medical University, Harbin, Heilongjiang Province 150081, China

## Abstract

**Background:**

The recombinant protein diannexin can inhibit platelet-mediated events, which contribute to acute respiratory distress syndrome (ARDS). Here, we investigated the effect of diannexin and its effect on heme oxygenase-1 (HO-1) in ARDS.

**Methods:**

A total of 32 rats were randomized into sham, ARDS, diannexin (D), and diannexin+HO-1 inhibitor (DH) groups. Alveolar-capillary permeability was evaluated by testing the partial pressure of oxygen to fraction of inspired oxygen (PaO_2_/FiO_2_) ratio, lung wet/dry weight ratio, and protein levels in the lung. Inflammation was assessed by measuring cytokine levels in the bronchial alveolar lavage fluid (BALF) and serum and nuclear factor-*κ*B (NF-*κ*B) in the lung tissue. Inducible nitric oxide synthase (iNOS), malondialdehyde (MDA), and myeloperoxidase (MPO) were measured to evaluate the oxidative stress response. Lung tissue pathology and apoptosis were also evaluated. We measured HO-1 expression in the lung tissue to investigate the effect of diannexin on HO-1 in ARDS.

**Results:**

Compared with the ARDS group, diannexin improved PaO_2_/FiO_2_, lung wet/dry weight ratio, and protein levels in the BALF and decreased levels of cytokines and NF-*κ*B in the lung and serum. Diannexin inhibited the oxidative stress response and significantly ameliorated pathological lung injury and apoptosis. The partial reversal of diannexin effects by a HO-1 inhibitor suggests that diannexin may promote HO-1 expression to ameliorate ARDS.

**Conclusions:**

We showed that diannexin can improve alveolar-capillary permeability, inhibit the oxidative stress response and inflammation, and protect against ARDS-induced lung injury and apoptosis.

## 1. Introduction

Acute respiratory distress syndrome (ARDS) is characterized by an increase in alveolar-vascular permeability, lung edema, and severe hypoxemia, leading to respiratory failure [1]. Mild, moderate, and severe ARDS have an incidence rate of approximately 30.0%, 46.6%, and 23.4%, respectively, as determined by intensive care unit data [2]. The mortality rate of ARDS ranges from 27% to 45% [1, 3]. Even mild ARDS has a mortality rate of approximately 30% [4]. ARDS is commonly treated with protective ventilation and pharmacological treatment, such as anti-inflammatory drugs and glucocorticoids, but the high mortality rate of ARDS suggests that these methods have poor clinical efficacy [2].

Considering the important role of platelets in inducing leukocyte adhesion in ARDS [5], platelet inhibition can significantly reduce ARDS [6, 7]. Annexin A5 can inhibit platelet activation [8] by reducing lipopolysaccharide (LPS) activity [9], thereby reducing vascular inflammation [10] and protecting against cardiac injury [11]. Unlike the short half-life of monomeric annexin A5, its recombinant homodimer diannexin has a longer half-life of 7 hours [12]. Diannexin can shield phosphatidylserine exposure and prevent cell adhesion, thereby improving blood flow [12], and can inhibit platelet accumulation [13] and alleviate multiple organ reperfusion injury [14–16].

Considering the important role of platelets in ARDS and the antiplatelet effect of diannexin, we speculated that diannexin can alleviate ARDS. As the effect of diannexin on ARDS has not been previously studied, we conducted this *in vivo* study using an ARDS rat model. Heme oxygenase-1 (HO-1) has previously been reported to protect against ARDS [17, 18], and since diannexin has been shown to upregulate HO-1 expression [15, 16], we also evaluated the effect of an HO-1 inhibitor (zinc protoporphyrin, ZNPP) in the present study to better understand the role of diannexin in ARDS.

## 2. Materials and Methods

### 2.1. Animals and Grouping

A total of 32 male Sprague Dawley rats (Harbin Medical University, China) were randomly assigned into four groups of 8 rats each: sham group, ARDS group, diannexin (D) group, and diannexin+HO-1 inhibitor (DH) group. All animal experiments in this study were performed with the approval from the Ethics Committee of Harbin Medical University, China.

### 2.2. Establishment of the ARDS Model

All rats were anesthetized with 30 mg/kg of 3% pentobarbital sodium via intraperitoneal injection. Briefly, a rat was placed into a plastic bag, and the skin on the back was clamped with the hand and the abdomen exposed. A 1 mL syringe was then inserted into the abdomen, and the anesthetic was injected. After the anesthesia was administered, tracheal intubation was performed with 16G catheter. While the rats were under local analgesia with 1% lidocaine, the caudal artery and vein were intubated with a 22G needle for blood sample collection and arterial blood analysis. Anesthesia and 0.5 mL saline were administered into the rats in the sham group via intratracheal instillation, while rats in the ARDS, D, and DH groups were administered 1 mg/kg of endotoxin (serotype 055:B5, Sigma-Aldrich, Israel) via the same method to induce ARDS [19]. Experimental ARDS animal models can be established by several methods, including intravenous or intratracheal LPS instillation. Given that clinical ARDS commonly results from local pulmonary infection that results in pneumonia, we opted for intratracheal LPS instillation to simulate ARDS and directly induce pneumonia in our rat model. Intravenous injections of 1 mL saline and 1 mg/kg diannexin were administered to rats as previously described [11, 14]. Rats in the DH group received 20 mg/kg of the HO-1 inhibitor, ZNPP (Sigma-Aldrich Chemical, USA) [20]. All rats were extubated and fed under sterile conditions for 24 h before anesthesia and intubation. All rats were injected with 0.6 mg/kg rocuronium before receiving mechanical ventilation (inspired oxygen fraction 50%, tidal volume (Vt) 10 mL/kg, respiratory rate of 50 breaths/min). After arterial blood analysis and blood sample collection, all rats were sacrificed via overdosage of anesthetics. The residual blood was flushed out with 4°C saline before opening the thorax via cutting the breast bone, and the heart and lungs were collected.

### 2.3. Blood Sample Collection

Arterial blood was collected to calculate the PaO_2_/FiO_2_ at baseline and at 24 h after endotoxin instillation. Peripheral blood was collected at the same time points to detect cytokine levels. The heart and lungs were collected after animal sacrifice. The right trachea was blocked with a vascular clamp, and 10 mL/kg saline was injected into the left lung tissue. After five washes of the saline, we collected the bronchoalveolar lavage fluid (BALF). Blood samples and BALF were centrifuged at 1,000 × g at 4°C for 15 min. The supernatants were collected and stored at -80°C for subsequent analyses.

### 2.4. Effect of Diannexin on Alveolar-Capillary Permeability

Alveolar-capillary permeability was estimated using the oxygen index, wet/dry weight ratio, and BALF protein levels. PaO_2_ was analyzed using the RapidLab 348 system (Bayer Diagnostics, Germany), and the oxygen index was calculated using the PaO_2_/FiO_2_ ratio. Part of the right upper lobe was weighed and dried at 60°C in a drying oven for 48 h and then weighed again to calculate the wet/dry weight ratio. BALF protein concentrations were measured using a BCA kit (Thermo Fisher, Shanghai, China).

### 2.5. Effect of Diannexin on Local and Systemic Inflammation

The effect of diannexin on inflammation was assessed by measuring BALF and serum cytokine levels. Intracellular adhesion molecule-1 (ICAM-1), macrophage inflammatory protein-1 (MIP-1), neutrophil elastase, tumor necrosis factor-*α* (TNF-*α*), interleukin (IL)-1*β*, and IL-8 were detected using ELISA kits (Wuhan Boster Bio-Engineering Limited Company, Wuhan, Hubei, China). We also counted the approximate number of neutrophils and macrophages in BALF deposits using Giemsa staining (Nikon 80i, Tokyo, Japan). Nuclear factor-*κ*B (NF-*κ*B) activity in the lung tissue was detected using the Transcription Factor Assay kit (Abcam, Toronto, Canada) according to the manufacturer's instructions. Briefly, nuclear proteins were extracted from lung tissues using the kit, and the extracted proteins were added into wells of the assay kit and incubated for 1 h before washing with wash buffer. The NF-*κ*B antibody was then added and incubated with the extracted proteins in the wells for 1 h before washing with wash buffer and subsequent incubation with the HRP-conjugated secondary antibody. The secondary antibody was then washed off, and the reaction mixture was added and incubated for 30 min. NF-*κ*B activity was analyzed using a microplate reader at 450 nm.

### 2.6. Effect of Diannexin on the Oxidative Stress Response

The lung tissue was homogenized with 0.9% saline (1 : 9 weight ratio) and then centrifuged at 1,000 × g for 15 min at 4°C to obtain the supernatant. We measured malondialdehyde (MDA) concentrations and myeloperoxidase (MPO) activity using respective kits (Nanjing Jiancheng, Nanjing, China). The expression of iNOS was also detected using western blot analysis.

### 2.7. Effect of Diannexin on Pathological Lung Injury

A part of the right lung tissue was fixed in paraformaldehyde and embedded in paraffin and then cut into 4 *μ*m sections to investigate the effect of diannexin on ARDS-induced histology. Hematoxylin and eosin (HE) staining was performed on the sections. The extent of pathological lung injury was evaluated by an independent pathologist blinded to the study. Pathology was determined using a previously described scoring system [21]. The following formula was used to calculate the score: score = [(20 × *A*) + (14 × *B*) + (7 × *C*) + (2 × *E*)]/(number of fields × 100).

### 2.8. Effect of Diannexin on Apoptosis in ARDS Rats

Apoptosis of ARDS-induced lung tissue injury was assessed using the TUNEL assay (Roche Diagnostics GmbH, Science, Mannheim, Germany). Briefly, each 4 *μ*m lung tissue section was immersed in proteinase K and then washed with phosphate-buffered saline (PBS). Next, the sections were first immersed in blocking buffer at room temperature for 10 min and then immersed in TUNEL reaction solution for 1 h at 37°C. After washing with PBS, endogenous peroxidase activity was inhibited by H_2_O_2_, and then, the sections were incubated in the extra-avidin peroxidase and diaminobenzidine solution. Subsequently, the lung tissue sections were counterstained with Mayer's hematoxylin, dehydrated, and mounted. An independent pathologist blinded to this study counted the number of apoptotic cells (brown nuclei) and total cells. The apoptosis index was calculated as the ratio of the number of apoptotic cells to total cells.

### 2.9. Western Blot Analysis

Proteins were extracted from lung tissues, and their concentrations were measured using the Bradford assay. Proteins were separated using a polyacrylamide gel followed by transfer to a polyvinylidene fluoride (PVDF) membrane. The PVDF membrane was incubated with primary antibodies including iNOS, HO-1, NF-*κ*B, Bax, and Bcl-xL (Sigma-Aldrich, St. Louis, MO, USA) overnight at 4°C, followed by incubation with the appropriate secondary antibodies (Santa Cruz Biotechnology, Oregon, USA). An enhanced chemiluminescence reagent (Santa Cruz Biotechnology Oregon, USA) was then added to visualize the proteins.

### 2.10. Effect of Diannexin on Coagulation

The prothrombin time (PT), activated partial thromboplastin (APTT), fibrinogen content (FIB), and platelet aggregation were tested using respective commercial coagulation assay kits (Steellex Biotechnology Co., Ltd., Taizhou, China) at 24 h after LPS injection to evaluate the effect of diannexin on coagulation in ARDS rats.

### 2.11. Statistical Analysis

Our preliminary calculation of the PaO_2_/FiO_2_ ratio (245 ± 30 mmHg) at 24 h after inducing ARDS in four rats indicated that at least 7 rats were necessary per group to obtain the target value of 300 mmHg (*α* = 0.05 and 1 − *β* = 90%). We randomly assigned 8 rats to each group. All data are presented as the mean ± standard deviation (SD), and statistical analysis was performed using the ANOVA test. Continuous data were analyzed using repeated measures ANOVA. All statistical analyses were performed using SPSS v19.0 for Windows (SPSS, Inc., IL, USA). Values of *P* < 0.05 were considered statistically significant.

## 3. Results

### 3.1. Diannexin Improves Alveolar-Capillary Permeability in ARDS Rats

**Figure 1:**
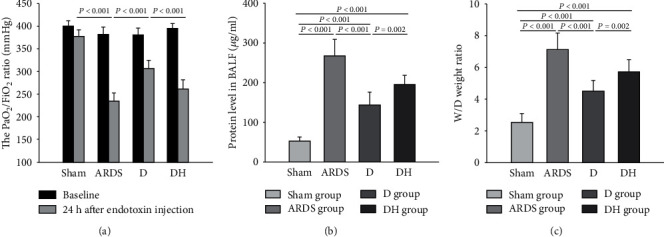
Diannexin improves the alveolar-capillary permeability. The rats in the sham group received saline instillation while rats in the ARDS, D, and DH groups received LPS instillation to induce ARDS. (a) Compared with the sham group, the oxygen index significantly decreased in all endotoxin-treated ARDS rats. Compared with the ARDS group, the D group showed a significantly increased oxygen index, but this effect was reversed by the HO-1 inhibitor in the DH group. (b) The total protein concentration in the BALF and the (c) wet/dry weight ratio in all ARDS rats were significantly higher compared to the sham group. These two indicators were ameliorated in the D group, but the cytoprotection conferred by diannexin was weakened by the HO-1 inhibitor in the DH group. Data are presented as the mean ± SD. Statistical analysis was performed using the ANOVA test.

Diannexin significantly decreased the PaO_2_/FiO_2_ ratio, total BALF protein concentration, and lung tissue wet/dry ratio in the ARDS group compared to the sham group (Figure 1). Notably, rats in the diannexin group showed a significantly higher PaO_2_/FiO_2_ ratio (sham *vs.* ARDS *vs.* D *vs.* DH: 378 ± 14*vs.*235 ± 18*vs.*307 ± 18*vs.*262 ± 20, *P* < 0.001; Figure 1(a)), total BALF protein concentration (sham *vs.* ARDS *vs.* D *vs.* DH: 52 ± 10*vs.*267 ± 41*vs.*143 ± 31*vs.*194 ± 23, *P* < 0.001; Figure 1(b)), and lung tissue wet/dry weight ratio (sham *vs.* ARDS *vs.* D *vs.* DH: 2.5 ± 0.5*vs.*6.8 ± 1.1*vs.*4.5 ± 0.6*vs.*5.2 ± 0.7, *P* < 0.001; Figure 1(c)) compared to the ARDS group. However, the presence of the HO-1 inhibitor ZNPP in the DH group appeared to counter the effect of diannexin and showed comparable effects to the ARDS group.

### 3.2. Diannexin Reduces Lung Injury and Apoptosis

**Figure 2:**
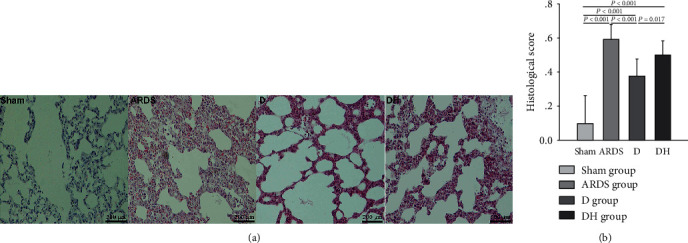
Diannexin reduces pathological lung injury in ARDS rats. (a) We observed typical pathological lung injury in ARDS rats. There was significant infiltration of inflammatory cells into the lung tissue. Significantly thickened and broken alveolar walls and severe edema were noted in the alveolar capillaries. Compared with the ARDS group, the D group showed improved alveolar morphology, but this improvement was partially blocked by the HO-1 inhibitor in the DH group. (b) Histogram showing the scores of the four study groups that reflect the extent of pathological lung damage as assessed by an independent pathologist. Data are presented as the mean ± SD. Statistical analysis was performed using the ANOVA test.

**Figure 3:**
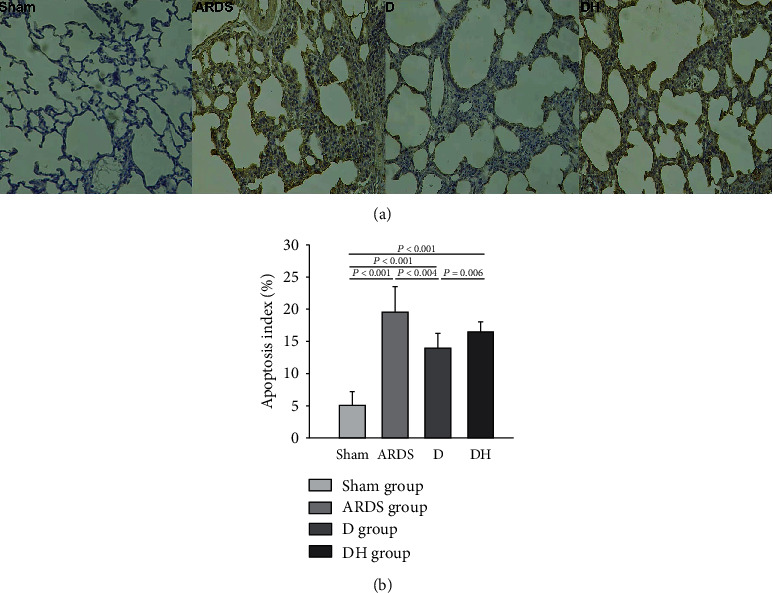
Diannexin decreases apoptosis. (a) Severe apoptosis was observed in all ARDS rats. The apoptotic cell count was decreased by diannexin in the D group, and the effect of diannexin was reversed by the HO-1 inhibitor in the DH group. (b) Histogram showing the apoptosis index of the four study groups. Data are presented as the mean ± SD. Statistical analysis was performed using the ANOVA test.

**Figure 4:**
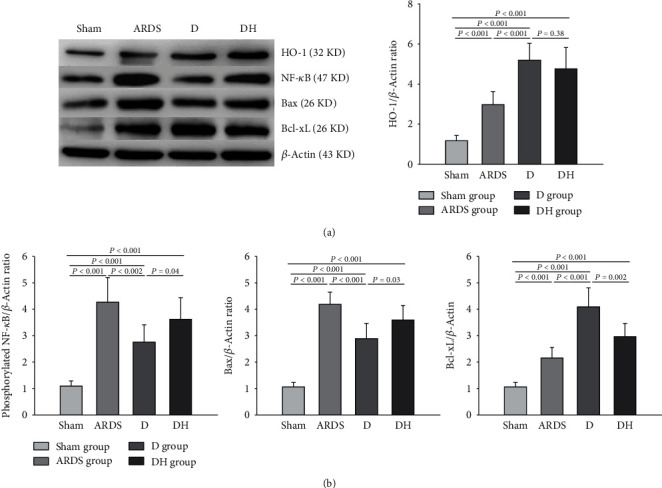
Diannexin regulates the level of proapoptotic and antiapoptotic proteins in the lung tissue. (a) Western blot showing the protein levels of HO-1, NF-*κ*B, Bax, and Bcl-xL. Administration of diannexin in the D group significantly reduced the level of proapoptotic protein Bax but increased the level of antiapoptotic protein Bcl-xL. The diannexin-induced effects were reversed by the HO-1 inhibitor in the DH group. (b) Histograms showing the relative levels of HO-1, NF-*κ*B, Bax, and Bcl-xL using *β*-actin as the internal reference. Data are presented as the mean ± SD. Statistical analysis was performed using the ANOVA test.

Histological examination of ARDS rats revealed severe lung damage, pulmonary and interstitial edema, alveolar collapse and breakage, and alveolar hemorrhage compared to the sham group (Figure 2). These adverse morphological changes were reduced in the D group but worsened with the addition of the HO-1 inhibitor as observed in rats of the DH group. We observed a significant increase in apoptosis in the ARDS and DH groups, but decreased apoptosis in the D group compared to the sham group (Figure 3). Apoptotic proteins were detected in lung tissues. In the D group, there was a decreased amount proapoptotic proteins and increased amount antiapoptotic proteins. This observation was reversed in the DH group, suggesting that ZNPP counteracts the cytoprotective effects of diannexin (Figure 4).

### 3.3. Diannexin Inhibits Local and Systemic Inflammation

**Figure 5:**
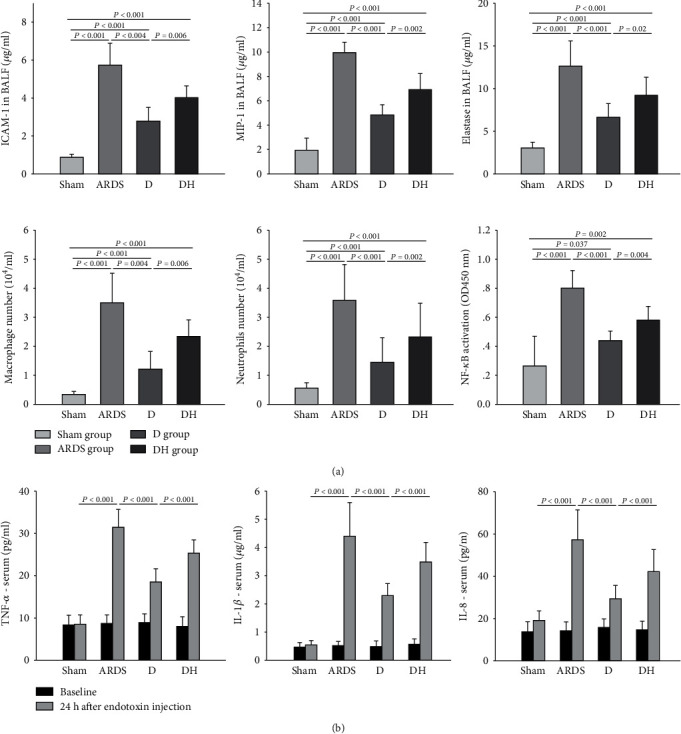
Diannexin reduces local and systemic inflammation in ARDS. Endotoxin-treated ARDS rats showed significantly higher levels of (a) proinflammatory factors (ICAM-1, MIP-1, elastase, macrophage count, neutrophil count, and NF-*κ*B activity) in the BALF and (b) serum (TNF-*α*, IL-1*β*, IL-8) compared to the sham group. Compared to the ARDS group, significantly decreased cytokine levels were observed in the D group, but the diannexin-mediated effects were inhibited by the HO-1 inhibitor in the DH group. Data are presented as the mean ± SD. Statistical analysis was performed using the ANOVA test.

We observed a significant increase in macrophage and neutrophil counts in the BALF at 24 h after inducing ARDS in rats compared to the sham group (Figure 5(a)). Diannexin significantly reduced the number of macrophages and neutrophils in the BALF compared to the ARDS group. However, addition of ZNPP in the DH group weakened the effect of diannexin on the number of inflammatory cells. A similar trend was observed for chemokine levels and neutrophil elastase in the BALF. There was a significant increase in ICAM-1 and MIP-1 chemokines and neutrophil elastase in the BALF in the ARDS group; the decreases in ICAM-1, MIP-1, and E-selectin levels in the D group were reversed in the DH group (Figure 5(a)). Furthermore, we found that TNF-*α*, IL-1*β*, and IL-8 levels were significantly upregulated in the serum of the ARDS group. Diannexin significantly reduced the cytokine levels in the serum of the D group, but the addition of ZNPP inhibited the effect of diannexin on these cytokines (Figure 5(b)). Rats in the D group also showed reduced phosphorylated NF-*κ*B levels and activity in the lung tissue, but these effects were reversed by ZNPP (Figures 4(a) and 5(a)).

### 3.4. Diannexin Reduces Oxidative Stress in the Lung Tissue

**Figure 6:**
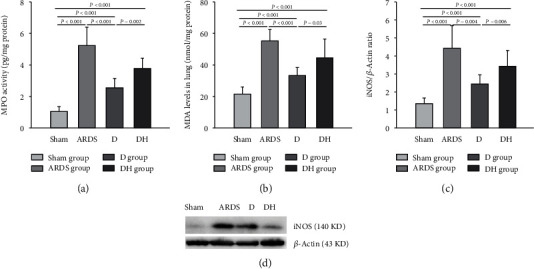
Diannexin inhibits the oxidative stress response in ARDS. A significantly higher oxidative stress response was observed in all ARDS rats compared to the sham group. (a) MPO activity, (b) MDA concentration, and (c) iNOS level were significantly decreased in the D group, but the antioxidative effects of diannexin were partially reversed by the HO-1 inhibitor. (d) Western blot showing the protein levels of iNOS . Administration of diannexin in the D group significantly reduced the level of iNOS. Data are presented as the mean ± SD. Statistical analysis was performed using the ANOVA test.

Diannexin significantly decreased the MPO activity and MDA and iNOS levels in the lung tissue in the ARDS group, which was countered by ZNPP in the DH group (Figure 6). These results indicate that ZNPP can inhibit the antioxidative effect of diannexin.

### 3.5. Diannexin Did Not Decrease Coagulation in ARDS Rats

**Figure 7:**
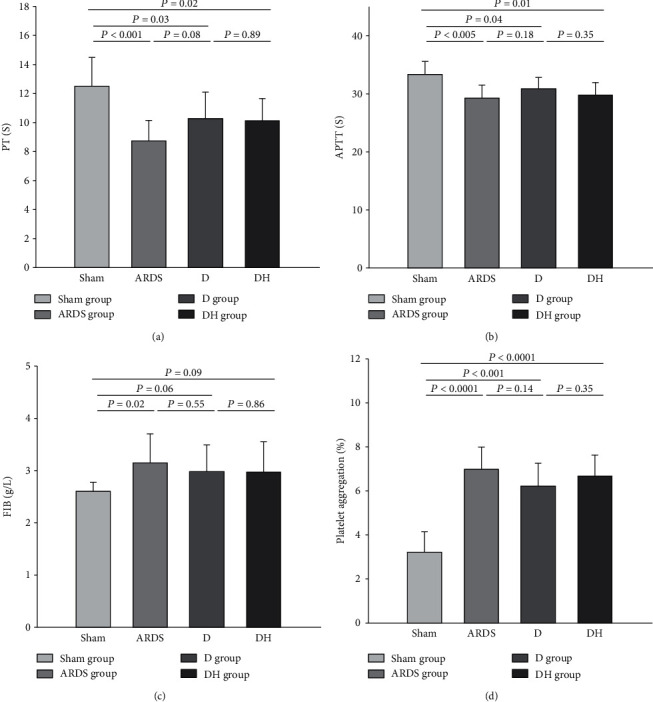
Diannexin does not affect coagulation in ARDS. Histograms showing the coagulation factors (a) PT, (b) APTT, (c) FIB, and (d) platelet aggregation in the four study groups. The ARDS rats showed higher coagulability compared to the sham group. Although the PT, APTT, FIB, and platelet activity were slightly changed in the D group compared to the ARDS group, the difference was not statistically significant. Data are presented as the mean ± SD. Statistical analysis was performed using the ANOVA test.

The ARDS group showed decreased PT and aPTT (Figures 7(a) and 7(b); *P* < 0.05) and increased FIB and platelet aggregation (Figures 7(c) and 7(d); *P* < 0.05) compared to the sham group. These indicators are consistent with the known occurrence of platelet activation and increased blood coagulation during ARDS. Elevated PT and aPTT and decreased FIB and platelet aggregation were observed in the D group compared to the ARDS group (Figures 7(a)–7(d); *P* > 0.05), indicating that diannexin lightly, but not significantly, reduces coagulation. Since the differences observed between the D and ARDS groups were not statistically significant, we cannot reliably assess the effect of ZNPP in the DH group.

## 4. Discussion

It was recently reported that the ARDS has an incidence rate of 10.4% in the ICU and a mortality rate ranging between 34.9% and 46.1% for mild to severe ARDS [2]. Platelet-mediated coagulation has been implicated in the development of ARDS, and inhibiting platelet-mediated coagulation could significantly attenuate lung injury [6, 7]. Notably, diannexin has been found to inhibit platelet-mediated effects [13], as well as attenuate various organ injuries [14–16]. Thus, we hypothesized that diannexin could play a critical protective role in ARDS and evaluate the effects of diannexin on disease progression in this study. We also examined diannexin's relationship with platelet coagulation in ARDS development using the HO-1 inhibitor ZNPP. We found that diannexin significantly improved the oxygen index and alveolar-capillary permeability, reduced pulmonary edema and pathological lung injury, and ameliorated inflammation and the oxidative stress response in our endotoxin-induced ARDS rat model. The weakening and partial reversal of diannexin effects by the HO-1 inhibitor suggest that diannexin can upregulate HO-1 to mediate protection against ARDS.

Inflammation plays a key role in ARDS development and progression. During ARDS, platelets are activated and interact with neutrophils to form platelet-neutrophil complexes, which significantly contribute to lung injury [22]. Proteins released from platelets can promote the secretion of proinflammatory factors from macrophages [23]. Activated platelets can also stimulate macrophages to produce extracellular traps, which aggravate inflammation [24]. Moreover, during ARDS, the damaged epithelia and endothelia will trigger NF-*κ*B activation and the release of chemokines including ICAM-1, MIP-1, and E-selectin, which in turn activate macrophages and neutrophils in the inflammatory response. In this study, the significant decrease in the number of neutrophils and macrophages in lung tissues and attenuated NF-*κ*B activation in the D group may be due to diannexin-induced inhibition of platelet-mediated events. Moreover, the observed decrease in the level of chemokines released from the endothelium and epithelium in the D group supports the anti-inflammatory effect of diannexin.

The oxidative stress response also plays an important role in ARDS development. During ARDS, NADPH oxidase in activated neutrophils converts oxygen into hydrogen peroxide and superoxide anions [25, 26]. Furthermore, iNOS activation in the damaged epithelia and endothelia promotes the production of excessive NO [27] and reactive nitrogen species (RNS), like peroxynitrite (ONOO-) [28], leading to lung injury. This can be ameliorated by inhibiting iNOS [29]. The final product of lipid peroxidation, MDA, is a direct indicator of the oxidative stress response [30]. In this study, we observed a significant reduction in MDA and iNOS levels in lung tissues from the D group. These results suggest that the antioxidant effect of diannexin conferred protection against ARDS. Indeed, other reports have shown that the antioxidant enzyme HO-1 could attenuate the oxidative stress response in lung injury by inhibiting the expression of iNOS and MPO, but increasing superoxide dismutase (SOD) levels. Consistent with previous studies, diannexin-mediated inhibition of the oxidative response observed in this study may be associated with increased HO-1.

In this study, we found the diannexin not only significantly reduced the release of TNF-*α* and Bax but also increased the expression of Bcl-xL. During ARDS, apoptotic endothelium and epithelium can significantly influence pulmonary function and patient outcomes. Inhibiting TNF-*α*, which promotes extrinsic apoptosis, can ameliorate apoptosis. The proapoptotic protein Bax promotes intrinsic apoptosis by activating Bid, which then activates caspase [31]. The antiapoptotic protein Bcl-xL can prevent the release of Bax from the mitochondria [32] and inhibit Bid activation [33], thereby preventing intrinsic and extrinsic apoptosis. Thus, our findings indicate a role of diannexin in inhibiting intrinsic and extrinsic apoptosis [31].

Since diannexin has strong antiplatelet property [13], it could induce severe complications like hemorrhage, which will worsen the outcome of ARDS. In this study, we noted increased coagulation in the ARDS group, as evidenced by lower PT and APTT and higher FIB compared to the sham group. No statistically significant difference in the coagulation indicators was observed in the D and DH groups compared to the ARDS group, suggesting that the dose of diannexin used in this study did not significantly influence coagulation. The apparent lack of effect of diannexin on coagulation could be due to the short half-life of diannexin of 7 hours that prevented its detection in the tests for these coagulation indicators. Indeed, the lack of effect of the dose of diannexin used in this study on coagulation is consistent with previous reports [14, 15, 34].

Although diannexin has been shown to reduce inflammation and attenuate multiple organ injury, the mechanism of diannexin has not been explored. Previous studies suggested that diannexin can promote the expression of HO-1 RNA in transplanted islets [15, 16]. Given the known anti-inflammatory and antioxidant effects of HO-1 in endotoxin-induced lung injury [35, 36], we speculated that the protective effects of diannexin in ARDS may be associated with HO-1 expression. Indeed, we showed that the HO-1 inhibitor weakened and partially reversed the effects of diannexin. These results suggest that diannexin may confer its protective effects in ARDS by upregulating HO-1 expression.

Despite extensive research and development of several therapies, there remains no effective cure for ARDS. The findings of this study offer a novel therapeutic strategy that could be employed in combination with other drugs, such as glucocorticoids, antibiotics, or protective ventilation, for patients with severe ARDS.

There are some limitations that should be noted in our study. First, it is important to consider that diannexin has antiplatelet activity that could induce hemorrhage. We did not examine the antiplatelet activity of diannexin in our study, and thus, there is uncertainty as to whether the dose of diannexin that we utilized could worsen coagulation. Although we did observe minimal impact on coagulation, the safe and effective dosage of diannexin should be confirmed in future follow-up studies. Another limitation of this study includes our focus on the effect of diannexin on ARDS induced by local infection. We did not consider other ARDS-causing factors including systemic infection, toxicity, or trauma induced by instillation, ventilation, or injury. The partial reversal of diannexin's effects by the HO-1 inhibitor ZNPP suggests the potential involvement of other unknown pathways, and future studies should examine these off-target effects of ZNPP. Cell experiments using ZNPP could be performed in future follow-up studies to confirm the effect of HO-1 on diannexin in ARDS and clarify the mechanism of action of diannexin.

## 5. Conclusions

Our findings show that diannexin significantly ameliorates ARDS lung injury by limiting inflammation, oxidation, and apoptosis. Our results demonstrate the association between diannexin and HO-1 in ARDS, which could potentially be exploited in ARDS therapy. Furthermore, the ARDS rat model established in the present study could serve as a valuable resource for future follow-up studies that investigate the mechanism of diannexin in ARDS.

## Data Availability

The datasets used and/or analyzed during the current study are available from the corresponding author on reasonable request.

## References

[1] Force A. D. T., Ranieri V. M., Rubenfeld G. D. (2012). Acute respiratory distress syndrome: the Berlin definition. *JAMA*.

[2] Bellani G., Laffey J. G., Pham T. (2016). Epidemiology, patterns of care, and mortality for patients with acute respiratory distress syndrome in intensive care units in 50 countries. *JAMA*.

[3] Sweeney R. M., McAuley D. F. (2016). Acute respiratory distress syndrome. *Lancet*.

[4] Pham T., Serpa Neto A., Pelosi P. (2019). Outcomes of patients presenting with mild acute respiratory distress syndrome: insights from the LUNG SAFE study. *Anesthesiology*.

[5] Silliman C. C., Voelkel N. F., Allard J. D. (1998). Plasma and lipids from stored packed red blood cells cause acute lung injury in an animal model. *The Journal of Clinical Investigation*.

[6] Grommes J., Alard J. E., Drechsler M. (2012). Disruption of platelet-derived chemokine heteromers prevents neutrophil extravasation in acute lung injury. *American Journal of Respiratory and Critical Care Medicine*.

[7] Chang Y. W., Tseng C. P., Lee C. H. (2018). *β*-Nitrostyrene derivatives attenuate LPS-mediated acute lung injury via the inhibition of neutrophil-platelet interactions and NET release. *American Journal of Physiology-Lung Cellular and Molecular Physiology*.

[8] Galan A. M., van Heerde W. L., Escolar G., Ordinas A., Sixma J., de Groot P. G. (2006). Antithrombotic action of annexin V proved as efficient as direct inhibition of tissue factor or thrombin. *European Journal of Clinical Investigation*.

[9] Rand J. H., Wu X. X., Lin E. Y., Griffel A., Gialanella P., McKitrick J. C. (2012). Annexin A5 binds to lipopolysaccharide and reduces its endotoxin activity. *mBio*.

[10] Ewing M. M., de Vries M. R., Nordzell M. (2011). Annexin A5 therapy attenuates vascular inflammation and remodeling and improves endothelial function in mice. *Arteriosclerosis, Thrombosis, and Vascular Biology*.

[11] de Jong R. C. M., Pluijmert N. J., de Vries M. R. (2018). Annexin A5 reduces infarct size and improves cardiac function after myocardial ischemia-reperfusion injury by suppression of the cardiac inflammatory response. *Scientific Reports*.

[12] Kuypers F. A., Larkin S. K., Emeis J. J., Allison A. C. (2007). Interaction of an annexin V homodimer (diannexin) with phosphatidylserine on cell surfaces and consequent antithrombotic activity. *Thrombosis and Haemostasis*.

[13] Rand M. L., Wang H., Pluthero F. G. (2012). Diannexin, an annexin A5 homodimer, binds phosphatidylserine with high affinity and is a potent inhibitor of platelet-mediated events during thrombus formation. *Journal of Thrombosis and Haemostasis*.

[14] Hashimoto K., Kim H., Oishi H. (2016). Annexin V homodimer protects against ischemia reperfusion-induced acute lung injury in lung transplantation. *The Journal of Thoracic and Cardiovascular Surgery*.

[15] Shen X. D., Ke B., Zhai Y. (2007). Diannexin, a novel annexin V homodimer, protects rat liver transplants against cold ischemia-reperfusion injury. *American Journal of Transplantation*.

[16] Cheng E. Y., Sharma V. K., Chang C. (2010). Diannexin decreases inflammatory cell infiltration into the islet graft, reduces *β*-cell apoptosis, and improves early graft function. *Transplantation*.

[17] Pereira M. L. M., Ortolan L. S., Sercundes M. K. (2016). Association of heme oxygenase 1 with lung protection in malaria-associated ALI/ARDS. *Mediators of Inflammation*.

[18] Yu J., Wang Y., Li Z. (2016). Effect of heme oxygenase-1 on mitofusin-1 protein in LPS-induced ALI/ARDS in rats. *Scientific Reports*.

[19] Rabelo M. A. E., Lucinda L. M. F., Reboredo M. M. (2018). Acute lung injury in response to intratracheal instillation of lipopolysaccharide in an animal model of emphysema induced by elastase. *Inflammation*.

[20] Wu S. Y., Li M. H., Ko F. C., Wu G. C., Huang K. L., Chu S. J. (2013). Protective effect of hypercapnic acidosis in ischemia-reperfusion lung injury is attributable to upregulation of heme oxygenase-1. *PLoS One*.

[21] Gustavo Matute-Bello G. D., Moore B. B., Groshong S. D. (2011). An official American Thoracic Society workshop report features and measurements of experimental acute lung injury in animals. *American Journal of Respiratory Cell and Molecular Biology*.

[22] Ortiz-Munoz G., Mallavia B., Bins A., Headley M., Krummel M. F., Looney M. R. (2014). Aspirin-triggered 15-epi-lipoxin A4 regulates neutrophil-platelet aggregation and attenuates acute lung injury in mice. *Blood*.

[23] Alfarsi M. A., Hamlet S. M., Ivanovski S. (2015). The effect of platelet proteins released in response to titanium implant surfaces on macrophage pro-inflammatory cytokine gene expression. *Clinical Implant Dentistry and Related Research*.

[24] Okubo K., Kurosawa M., Kamiya M. (2018). Macrophage extracellular trap formation promoted by platelet activation is a key mediator of rhabdomyolysis-induced acute kidney injury. *Nature Medicine*.

[25] Sato K., Kadiiska M. B., Ghio A. J. (2002). In vivo lipid-derived free radical formation by NADPH oxidase in acute lung injury induced by lipopolysaccharide: a model for ARDS. *The FASEB Journal*.

[26] Liu Y. Y., Li L. F., Fu J. Y. (2014). Induced pluripotent stem cell therapy ameliorates hyperoxia-augmented ventilator-induced lung injury through suppressing the Src pathway. *PLoS One*.

[27] Peng X., Abdulnour R. E., Sammani S. (2005). Inducible nitric oxide synthase contributes to ventilator-induced lung injury. *American Journal of Respiratory and Critical Care Medicine*.

[28] Johnson E. R., Matthay M. A. (2010). Acute lung injury: epidemiology, pathogenesis, and treatment. *Journal of Aerosol Medicine and Pulmonary Drug Delivery*.

[29] Zheng H., Liang W., He W. (2019). Ghrelin attenuates sepsis-induced acute lung injury by inhibiting the NF-*κ*B, iNOS, and Akt signaling in alveolar macrophages. *American Journal of Physiology. Lung Cellular and Molecular Physiology*.

[30] Liu K. X., Wu W. K., He W., Liu C. L. (2007). Ginkgo biloba extract (EGb 761) attenuates lung injury induced by intestinal ischemia/reperfusion in rats: roles of oxidative stress and nitric oxide. *World Journal of Gastroenterology*.

[31] Cartron P. F., Juin P., Oliver L., Meflah K., Vallette F. M. (2003). Impact of proapoptotic proteins Bax and Bak in tumor progression and response to treatment. *Expert Review of Anticancer Therapy*.

[32] Niture S. K., Jaiswal A. K. (2013). Nrf2-induced antiapoptotic Bcl-xL protein enhances cell survival and drug resistance. *Free Radical Biology & Medicine*.

[33] Wang X., Zhang J., Kim H. P., Wang Y., Choi A. M., Ryter S. W. (2004). Bcl-XL disrupts death-inducing signal complex formation in plasma membrane induced by hypoxia/reoxygenation. *FASEB Journal*.

[34] (2006). Abstracts of the 57th annual meeting of the American Association for the Study of Liver Diseases, October 27-31, 2006, Boston, Massachusetts, USA. *Hepatology*.

[35] Yu J., Shi J., Wang D. (2016). Heme oxygenase-1/carbon monoxide-regulated mitochondrial dynamic equilibrium contributes to the attenuation of endotoxin-induced acute lung injury in rats and in lipopolysaccharide-activated macrophages. *Anesthesiology*.

[36] Park J., Chen Y., Zheng M. (2018). Pterostilbene 4-*β*-Glucoside Attenuates LPS-Induced Acute Lung Injury via Induction of Heme Oxygenase-1. *Oxidative Medicine and Cellular Longevity*.

